# One-Step Carbonization Synthesis of Magnetic Biochar with 3D Network Structure and Its Application in Organic Pollutant Control

**DOI:** 10.3390/ijms232012579

**Published:** 2022-10-20

**Authors:** Xiaoxin Chen, Jiacheng Lin, Yingjie Su, Shanshan Tang

**Affiliations:** 1 College of Life Sciences, Jilin Agricultural University, Changchun 130118, China; 2 Key Laboratory of Straw Comprehensive Utilization and Black Soil Conservation, Ministry of Education, Jilin Agricultural University, Changchun 130118, China

**Keywords:** water, biochar, organic pollutants, adsorption, Fenton-like catalysis

## Abstract

In this study, a magnetic biochar with a unique 3D network structure was synthesized by using a simple and controllable method. In brief, the microbial filamentous fungus *Trichoderma reesei* was used as a template, and Fe^3+^ was added to the culture process, which resulted in uniform recombination through the bio-assembly property of fungal hyphae. Finally, magnetic biochar (BMFH/Fe_3_O_4_) was synthesized by controlling different heating conditions in a high temperature process. The adsorption and Fenton-like catalytic performance of BMFH/Fe_3_O_4_ were investigated by using the synthetic dye malachite green (MG) and the antibiotic tetracycline hydrochloride (TH) as organic pollutant models. The results showed that the adsorption capacity of BMFH/Fe_3_O_4_ for MG and TH was 158.2 and 171.26 mg/g, respectively, which was higher than that of most biochar adsorbents, and the Fenton-like catalytic degradation effect of organic pollutants was also better than that of most catalysts. This study provides a magnetic biochar with excellent performance, but more importantly, the method used can be effective in further improving the performance of biochar for better control of organic pollutants.

## 1. Introduction

As one of Earth’s natural resources, water is essential for all aspects of life. Except in various forms in the oceans, glaciers, and the atmosphere, less than 0.3 per cent of the water on Earth is fresh enough to meet the survival and development needs of all living things [1]. Furthermore, nearly one-fifth of the world’s population still does not have access to safe drinking water [2]. Therefore, water safety has always been a major, widespread issue. With the rapid development of society, the production and discharge of a large number of industrial processes containing various dyes, drugs, heavy metals, and other harmful wastewater has gradually become one of the main risks threatening the safety of water bodies [3]. Among them, organic pollutants significantly reduce water quality, and in addition to affecting ecosystems by disrupting photosynthesis of aquatic plants, they also have a significant impact on human health [4,5]. Studies have pointed out that most organic pollutants are persistent forms of pollution (synthetic dyes, antibiotics, etc.), which are difficult to degrade naturally, are toxic and carcinogenic, have high tolerance, and have begun to threaten the global water environment [6,7]. As highlighted by the United Nations Sustainable Development Goals, providing clean water remains a major challenge, especially in developing regions of the world [8], so the need for more efficient and advanced sewage purification technologies is becoming more urgent.

Bio-remediation [9], membrane treatment [10], precipitation [11], microbial degradation [12], and other technologies have been used to remove pollutants from water, but most of the treatment technologies often make it difficult to effectively and thoroughly treat these organic pollutants. Adsorption and catalysis technologies have attracted extensive attention because of their low cost, easy operation, safety, and control [13]. Adsorption is a surface phenomenon where organic contaminants adhere to the surface of an adsorbent by physical or chemical attraction, whereas catalysis refers to the acceleration of chemical conversion of pollutants by the addition of catalysts [14,15]. The traditional catalytic treatment of pollutants can be divided into photo-catalysis and electro-catalysis; the former requires expensive precious metals (such as Ti or Ag), while the latter requires a lot of electricity. These disadvantages have made these methods less attractive for the treatment of pollutants [16,17]. Conversely, the Fenton/Fenton-like catalytic process is one of the most effective and promising advanced catalytic processes due to its low cost, mild operating conditions, and easy recovery [18,19,20]. In addition, iron-based catalysts have gradually become a research hotspot due to their abundant resources, environmental friendliness, and high efficiency. In recent years, it has become an effective method for loading or embedding iron-based nanoparticles with biochar to form magnetic biochar materials [21,22]. On the one hand, the loading of iron-based nanoparticles means that biochar can easily be recycled by magnets. On the other hand, biochar materials can maintain a high concentration of local pollutants through adsorption, thus improving the catalytic efficiency of iron-based catalysis.

In order to pursue the extreme performance of materials so as to better cope with water security crises, many scientists have developed a large number of materials with unique structures and excellent performance [23,24]. Among many advanced materials, biochar has received much attention because of its attractive structure, huge specific surface area, rich functional groups, and so on [25,26,27]. In general, the characteristics and properties of biochar often determine its application. Among them, the main parameters affecting biochar should include physicochemical properties, performance, production process, and cost factors. Its physical and chemical properties and properties depend on the synthesis technology (that is, the production process), and the source of carbon and more advanced processes are the main basis for reducing the production cost. Therefore, how to develop new and cheaper carbon sources to synthesize biochar materials with higher performance through simpler or more efficient processes is the focus of many researchers. Research into the production of biochar from cheaper and more readily available biomass is, of course, important, but the development of new carbon sources is also essential. Recently, micro-sized biochar materials based on microorganisms such as bacteria, fungi, and viruses have become one of the most popular hotspots [28]. Due to their strong life activity, high reproduction rate, unique structure, and cheap source, microorganisms, especially fungal hyphae, are widely present in nature as one of the important carbon resources used to prepare biochar materials [29]. Fungal hypha is the structural unit of most fungi, which is formed by the continuous division of fungal spores after germination, finally forming a three-dimensional (3D) network structure [30,31]. This fascinating 3D network is often used as a template for biomass composites, which provides a great motivation for us to continue exploring this interesting resource.

In this work, *Trichoderma reesei* (*T. reesei*), a filamentous fungus which has short growth cycles and large fungal hyphae (FH) volumes, was used as a template to support the micro-sized 3D network structure. In the process of fungal culture, Fe^3+^ can complete uniform biological assembly with the FH through the characteristics of step-wise growth. Then, a magnetic biochar with a 3D network structure (BMFH/Fe_3_O_4_) was prepared by fractional heating. Here, the presence of magnetic nanoparticles not only makes it easier to recover biochar from water but also endows biochar with the effect of Fenton-like catalytic reagents, thus further improving the treatment effect of pollutants. Synthesized BMFH/Fe_3_O_4_ can be used as the ideal biochar materials for removing organic pollutants including malachite green (MG) and tetracycline hydrochloride (TH). The adsorption performance of BMFH/Fe_3_O_4_ on organic pollutants was investigated by batch adsorption experiments, including concentration, pH, and temperature. Subsequently, H_2_O_2_ was used to construct a heterogeneous Fenton-like system to effectively catalyze the degradation of organic pollutants. Moreover, the stability and reusability of the BMFH/Fe_3_O_4_ were also investigated. The focus of this study is to develop a novel strategy for the synthesis of magnetic biochar materials through a one-step process of biological assembly and fractional heating for the effective removal of organic pollutants from water.

## 2. Results and Discussion

### 2.1. Preparation of BMFH/Fe_3_O_4_

**Figure 1:**
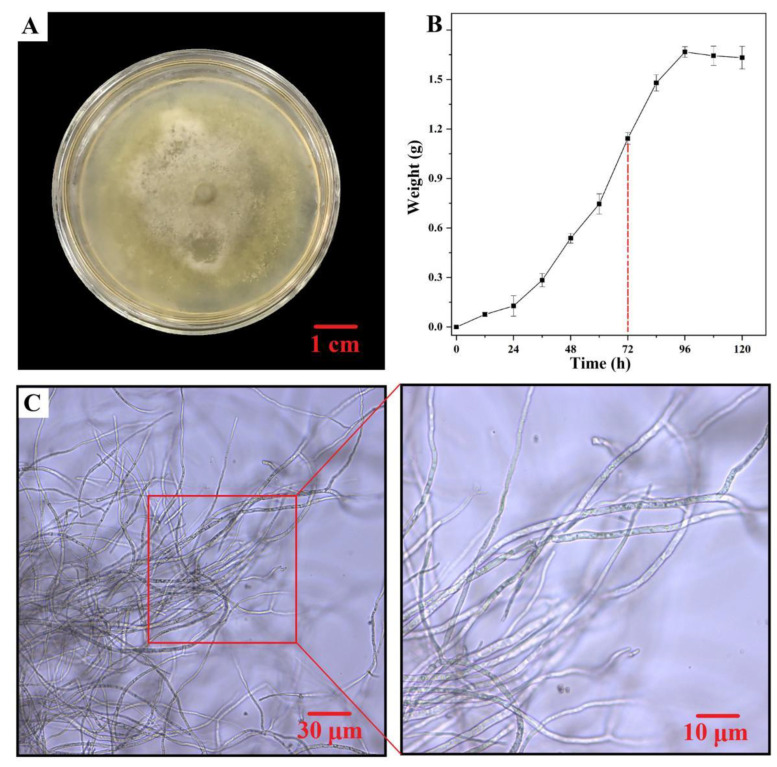
Biological morphology (**A**), growth curve test (**B**), and optical microscope structure observation results (**C**) of *T. reesei*.

*T. reesei* is a multicellular eukaryotic microorganism, the asexual form of *Hypocrea jecorina*, belonging to the genus *Penicillium* (*Moniliales*), which is a typical filamentous fungus [32,33]. *T. reesei* was cultured on PDA plate medium to observe the colony biomorphology during its growth, and the results are shown in Figure 1A. *T. reesei* filaments presented white dense flat hyphae at the initial stage of growth, and then became white flocculent, and grew diffusely to the periphery. With the progress of culture, a pale-green spore-producing cluster area appeared at the edge of the colony. The growth curve of *T. reesei* was measured by the dry weight method through liquid PDL culture medium, and the results are shown in Figure 1B. It can be seen that *T. reesei* began to enter the logarithmic growth phase at 24 h, the fastest growth was achieved at 72 h, and it entered the stable phase after 96 h. The complete culture of *T. reesei* was placed under a light microscope to observe its mycelial morphology, and the results are shown in Figure 1C. We can see that *T. reesei* is composed of hyphae with diameters of 1–5 um, which interweave and intertwine with each other, presenting a classical 3D network structure, indicating that *T. reesei* has great potential as a carbon source for high-performance biochar materials.

**Scheme 1:**
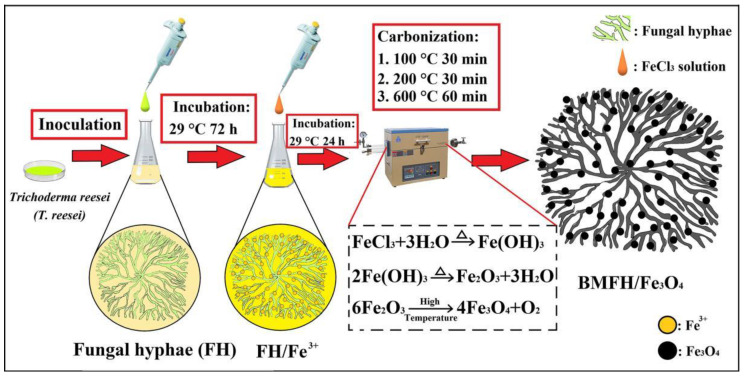
Schematic plot of the preparation of BMFH/Fe_3_O_4_.

The preparation process of BMFH/Fe_3_O_4_ is shown in Scheme 1 and can be divided into two stages. First, the filamentous fungus *T. reesei* was inoculated in a triangle flask containing PDL medium and incubated in a constant temperature shaker (29 °C, 150 RPM). The shear force of the liquid maintains the continuous growth of mycelia, thus forming a stable 3D network structure. Fe^3+^ was added when the mycelium grew the fastest (72 h), and the Fe^3+^ combined evenly with the mycelium by shaking the flask to form a load. After culture (96 h), the mycelium was collected and washed with ultrapure water to remove excess Fe^3+^ on the surface. Finally, Fe^3+^ loaded and uniformly dispersed mycelial composite FH/Fe^3+^ was obtained. Then, in order to obtain magnetic BMFH/Fe_3_O_4_, the obtained FH/Fe^3+^ was heated at a high temperature to complete the carbonization and compound magnetic process. In the process of this one-step synthesis method, we controlled the final product by dividing the heating into three stages (100 °C for 30 min, 200 °C for 30 min, and 600 °C for 60 min). In the first stage, when the temperature reached 100 °C, the moisture adsorbed by the mycelium evaporated due to heat, and at this time, Fe^3+^ reacted with water vapor to form ferric hydroxide colloid (Fe(OH)_3_). As the temperature rose above 200 °C, the colloidal ferric hydroxide was thermally decomposed to form ferric oxide (Fe_2_O_3_) and water (H_2_O). At this time, the water vapor and the volatile substances formed by the decomposition of oxygenated components during the thermal lysis of mycelium were simultaneously discharged from the reaction system. When the temperature continued to rise to 600 °C, the carbonation process of mycelium continued, and the iron oxide decomposed into ferric tetroxide (Fe_3_O_4_) magnetic nanoparticles due to a high-temperature reaction. Finally, the magnetic mycelial biochar BMFH/Fe_3_O_4_ with 3D network structure after carbonization and composite magnetism was successfully obtained.

### 2.2. Characterization Results

**Figure 2:**
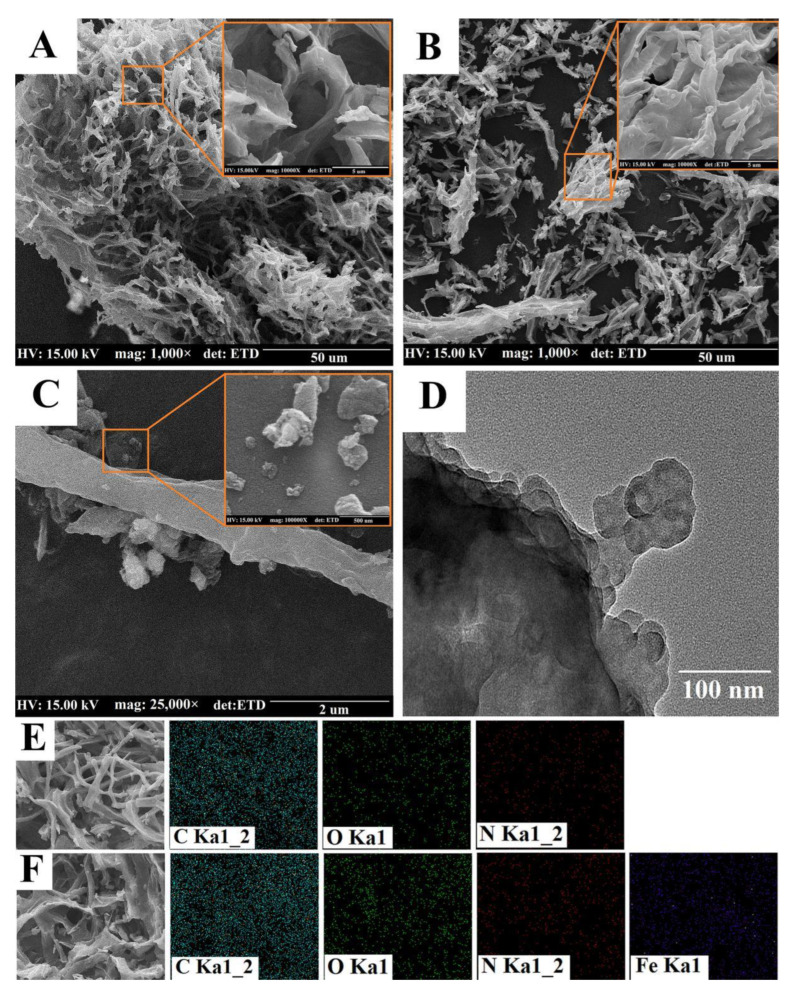
SEM images of (**A**) BMFH, (**B**) BMFH/Fe_3_O_4_-0.01, and (**C**) Fe_3_O_4_ nanoparticles loaded on the BMFH. (**D**) TEM image of BMFH/Fe_3_O_4_-0.01. EDS mapping of (**E**) BMFH and (**F**) BMFH/Fe_3_O_4_-0.01.

The morphologies of the samples were characterized by SEM, TEM, and EDS, as shown in Figure 2. It is clearly seen from Figure 2A that many slender hyphae are interwoven to form this fascinating 3D network. BMFH, which is made from carbonized mycelia, still retains its structure, but its constituent mycelia are much finer than those without carbonization (Figure 1C), indicating that the carbonization process is quite complete and successful. However, after adding Fe^3+^, BMFH/Fe_3_O_4_ also has a 3D network structure, but it does appear to be somewhat fragmented compared with BMFH. This may be because the preparation process involves the synthesis of Fe_3_O_4_ at a high temperature. During this process, a large amount of water vapor and oxygen are released during the generation of magnetic nanoparticles, which causes the carbonation process to become more violent, resulting in the fracture of a large number of mycelial structures. In addition, as seen in Figure 2C,D, a certain amount of obvious Fe_3_O_4_ nanoparticles was successfully loaded onto FH, which demonstrated the feasibility and effectiveness of the one-step method. Energy dispersive spectrometer (EDS) was used to analyze the elemental composition of samples, and the results are shown in Figure 2E,F and Table S1 (Supplementary Materials). Clearly, BMFH mainly comprises C (83.16%), O (14.63%), and N (2.21%) elements (Figure 2E and Table S1). There was also a large amount of Fe on the surface of BMFH/Fe_3_O_4_ compared with BMFH, which once again proved that the one-step method can be used to synthesize Fe_3_O_4_ and load magnetic nanoparticles onto the surface of mycelium through the carbonization process.

**Figure 3:**
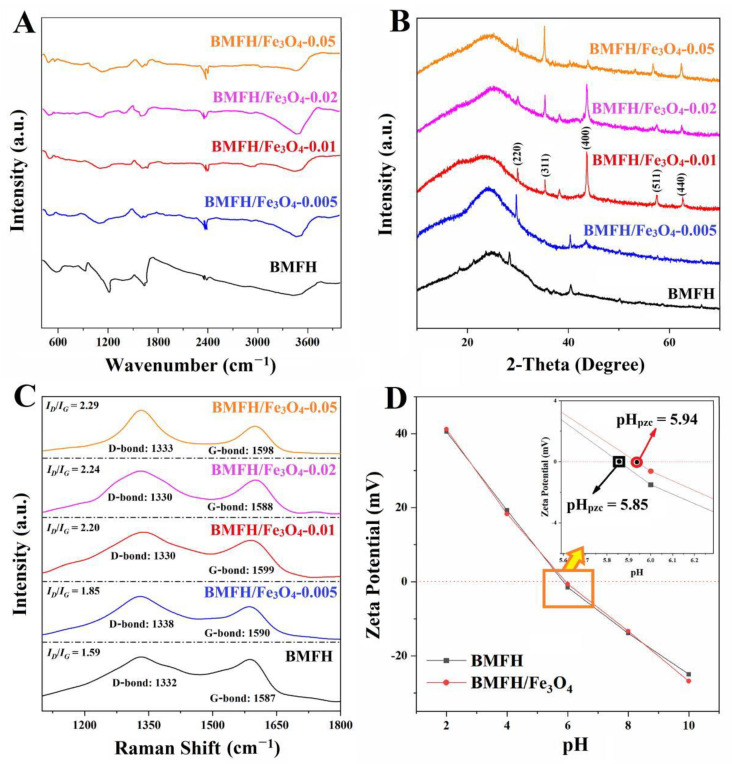
(**A**) FT-IR spectra, (**B**) XRD pattern, (**C**) Raman spectra of samples, and (**D**) Zeta potential tests of BMFH/Fe_3_O_4_-0.01.

The functional groups of the samples were analyzed by FT-IR, as shown in Figure 3A. The wide band corresponding to the stretching vibration of the hydroxyl functional group at a range of 3400–3500 cm^−1^ indicates that a large number of oxygen-containing functional groups [23,24,25,26,27] exist on all samples. In the range of 2800–2900 cm^−1^, symmetric and asymmetric tensile vibration peaks of -CH groups were found [30,31], which may be caused by the thermal cracking of macromolecules from FH at high temperature and the removal of a large amount of hydrogen [31,34]. Bands in the range of 1600–1700 cm^−1^ represent C=C stretching of the aromatic ring, which can be interpreted as the decomposition of the C-H bond into a more stable aromatic C=C bond after high-temperature treatment [31]. The overlapping band in the 900–1300 cm^−1^ region corresponds to the stretching vibration of C-O and C-N heterocycles [31,34]. The band at 600–750 cm^−1^ in BMFH represents SiO_2_ [25]. Interestingly, after Fe^3+^ compound is formed, the band there becomes weak and even disappears gradually compared with the BMFH/Fe_3_O_4_ samples. The characteristic band at 470–570 cm^−1^ representing the Fe-O group from Fe_3_O_4_ [25] can be observed, which means the Fe_3_O_4_ particles were successfully loaded onto the BMFH.

The crystal structures of the samples were analyzed by XRD, as shown in Figure 3B. BMFH and BMFH/Fe_3_O_4_ prepared with different concentrations of Fe^3+^ were both amorphous structures. The peaks at 19, 27, and 40^°^ of BMFH were related to inorganic salts, which were observed probably due to the salting-out effect by water loss or cracking of FH during the high-temperature pyrolysis [24]. From all the BMFH/Fe_3_O_4_ samples, we can clearly see that there are many characteristic peaks, such as (220), (311), (400), (511), and (440), which belong to Fe_3_O_4_ with a classical structure [25]. It is worth noting that the Fe^3+^ concentration used in the preparation of BMFH/Fe_3_O_4_-0.005 is the lowest, so the characteristic peak signal of Fe_3_O_4_ is the weakest in the prepared samples, with many peak positions not even capable of being easily captured compared with BMFH/Fe_3_O_4_-0.01.

Defects in carbon-based materials are usually caused by atomic losses or lattice distortions; here, Raman spectra were used to test the BMFH and BMFH/Fe_3_O_4_ samples, as shown in Figure 3C. Two typical characteristic peaks were the D-band associated with amorphous carbon, approximately 1334 ± 4 cm^−1^, and the G-band associated with graphitic carbon, approximately 1593 ± 6 cm^−1^ [24,25,26]. The intensity ratio of D-band (*I_D_*) and G-band (*I_G_*) is an important parameter to measure the degree of defect and disorder in carbon, shown as *I_D_*/*I_G_*. The increase in *I_D_*/*I_G_* values of all BMFH/Fe_3_O_4_ samples (1.85 for BMFH/Fe_3_O_4_-0.005, 2.20 for BMFH/Fe_3_O_4_-0.01, 2.24 for BMFH/Fe_3_O_4_-0.02, and 2.29 for BMFH/Fe_3_O_4_-0.05) compared with BMFH (1.59) indicates the presence of more amorphous structures in the biochar material, either as amorphous carbon or due to Fe_3_O_4_. 

A zeta potential test was used to evaluate the surface charges of BMFH and BMFH/Fe_3_O_4_-0.01, as shown in Figure 3D. Like many biochar materials [29,30,31], when pH changes from acid to basic (2 to 10), the surface charges of samples change from positive (40.5 and 41.2 for BMFH and BMFH/Fe_3_O_4_-0.01, respectively) to negative (−25.0 and −26.8 for BMFH and BMFH/Fe_3_O_4_-0.01, respectively). When the pH value is lower than pH_pzc_, the sample surface is positively charged, and vice versa. The pH_pzc_ of BMFH is 5.85, which is lower than BMFH/Fe_3_O_4_-0.01 (5.94), indicating that Fe_3_O_4_ loaded onto the samples may have a slight effect on the charge of magnetic biochars. The pH_pzc_ of all samples was less than 7.00, indicating that they are more suitable for the water treatment in an alkaline environment.

**Figure 4:**
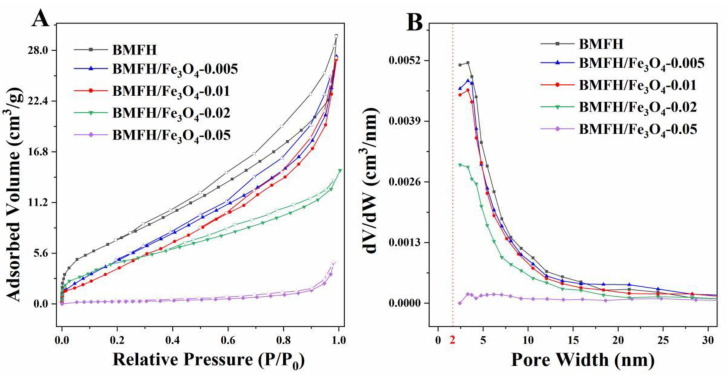
(**A**) N_2_ adsorption–desorption isotherms and (**B**) pore size distribution curves of BMFH, BMFH/Fe_3_O_4_-0.005, BMFH/Fe_3_O_4_-0.01, BMFH/Fe_3_O_4_-0.02, and BMFH/Fe_3_O_4_-0.05.

N_2_ adsorption–desorption isotherms were used to evaluate the specific surface area (S_BET_) and pore structure of biochar materials, as shown in Figure 4 and Table 1. Both BMFH and BMFH/Fe_3_O_4_ prepared under different conditions exhibited typical type IV isotherms with H3 hysteresis loop (Figure 4A), which indicated that they were both typical mesoporous materials [29,30]. The specific surface area and the total pore volume of BMFH were 27.91 m^2^/g and 0.0369 cm^3^/g, respectively. With the increase in Fe^3+^ concentration in the preparation of BMFH/Fe_3_O_4_, the specific surface area decreased significantly. When the concentration of Fe^3+^ increased to 0.05 M (BMFH/Fe_3_O_4_-0.05), the specific surface area sharply decreased to 0.89 m^2^/g. At this time, the pore volume of BMFH/Fe_3_O_4_-0.05 was 0.0071cm^3^/g, which can be said to have lost most of the pore structure compared with BMFH. Figure 4B shows that the mean pore sizes of BMFH and magnetic biochar range from 2 to 30 nm, which again indicate that they are mesoporous materials. In addition, the adsorption capacities of BMFH and magnetic biochars prepared under different conditions for organic pollutants (MG and TH) were compared (Figure S3, Supplementary Materials). The results showed that the adsorption capacity was positively correlated with the specific surface area and total pore volume to some extent [31]. Interestingly, there was no significant difference in adsorption capacity and specific surface area between BMFH/Fe_3_O_4_-0.01 and BMFH/Fe_3_O_4_-0.005. Therefore, on the basis of comprehensive considerations, BMFH/Fe_3_O_4_-0.01 was selected as the model representative of magnetic biochar in order to facilitate subsequent studies (adsorption, Fenton-like catalysis, and cyclic stability studies).

**Figure 5:**
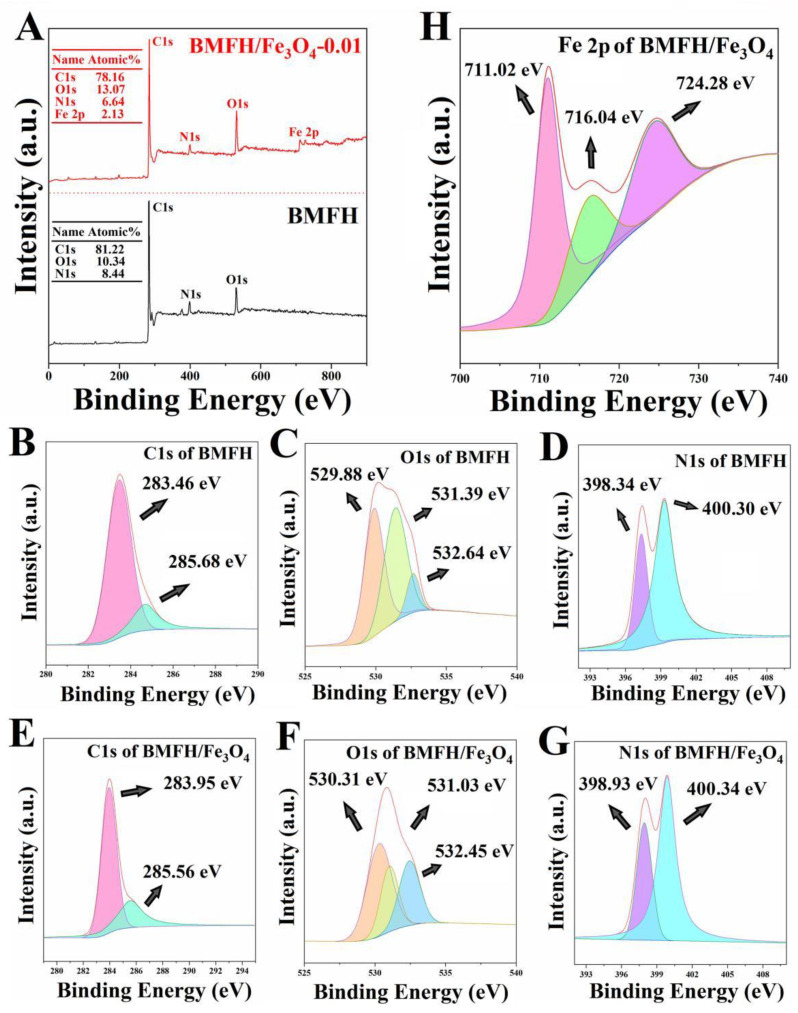
(**A**) XPS spectra of BMFH and BMFH/Fe_3_O_4_. The (**B**) C1s, (**C**) O1s, and (**D**) N1s of BMFH. The (**E**) C1s, (**F**) O1s, (**G**) N1s, and (**H**) Fe 2p of BMFH/Fe_3_O_4_.

The surface chemical properties and electronic states of the BMFH and BMFH/Fe_3_O_4_-0.01 were analyzed by XPS, as shown in Figure 5. It can be seen that BMFH contains mainly C (81.22%), O (10.34%), and N (2.13%) elements, and BMFH/Fe_3_O_4_-0.01 contains C (71.86%), O (13.07%), N (6.64%), and Fe (2.13) elements. The C/O ratio of BMFH/Fe_3_O_4_-0.01 (5.98) is significantly lower than that of BMFH (7.85), indicating that loaded Fe_3_O_4_ introduced more O element, which also proved the reliability of this one-step method. The high-resolution C1s spectrum of the BMFH (Figure 5B) and BMFH/Fe_3_O_4_-0.01 (Figure 5E) showed two peaks, including C-C at approximately 283.70 ± 0.25 eV and C-O at approximately 285.62 ± 0.06 eV [23,24]. The high-resolution O1s spectrum of the BMFH (Figure 5C) and BMFH/Fe_3_O_4_-0.01 (Figure 5F) showed three peaks, including the quinones at approximately 530.10 ± 0.22 eV, C=O at approximately 531.21 ± 0.18 eV, and C-O at approximately 532.55 ± 0.10 eV [24,26]. The high-resolution N1s spectrum of the BMFH (Figure 5D) and BMFH/Fe_3_O_4_-0.01 (Figure 5G) showed two peaks: pyridinic-N at approximately 398.64 ± 0.30 eV and pyrrolic-N at approximately 400.32 ± 0.02 eV [27]. The high-resolution Fe 2p spectrum of BMFH/Fe_3_O_4_-0.01 (Figure 5H) showed three peaks: Fe 2p_3/2_ corresponding to 711.02 eV, Fe 2p_1/2_ corresponding to 724.28 eV, and shakeup satellite peak at approximately 716.04 eV, which revealed the characteristic peaks of Fe in Fe_3_O_4_ [25]. On the basis of all the above data, BMFH/ Fe_3_O_4_-0.01 was selected as the model BMFH/Fe_3_O_4_ for subsequent study.

### 2.3. Adsorption Performances

#### 2.3.1. Adsorption Kinetic

**Figure 6:**
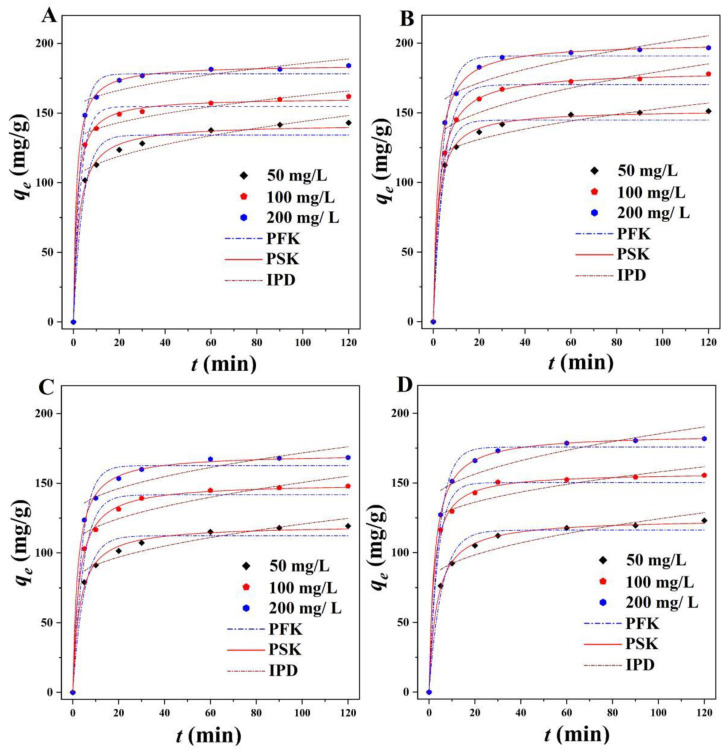
PFK, PSK, and IPD plots of BMFH (**A**,**B**) and BMFH/Fe_3_O_4_ (**C**,**D**) for MG and TH at 303 K.

Adsorption kinetics is often used to study the influence of the adsorbate concentration on the adsorption process and the control mechanism of chemical reactions in this process. Generally, the study of adsorption kinetics can help us to better understand the behavior of adsorbent in the adsorption process and help to study the mechanism of this process [14]. The effect of contact time on the adsorption of MG and TH (50, 100, and 200 mg/L) by BMFH and BMFH/Fe_3_O_4_ is shown in Figure 6. It can be seen that all the adsorption processes have the same trend. The first is a rapid adsorption process that dramatically increases the adsorption capacity in the first 10 min and gradually reaches adsorption saturation in 60 min. In addition, the adsorption capacity increases with the increase in the initial concentration of adsorbed substance. These results indicate that both BMFH and BMFH/Fe_3_O_4_ have great potential to remove organic pollutants (MG and TH).

Models that control surface adsorption are defined as adsorption reactions and empirical models including the pseudo-first-order kinetic (PFK, Equation (1)), pseudo-second-order kinetic (PSK, Equation (2)), and the intra-particle diffusion model (IPD, Equation (3)). The models are expressed below:

(1) $$q_{t}=q_{e}-\frac{q_{e}}{e^{k_{1}t}}$$

(2) $$q_{t}=\frac{k_{2}q_{e}{}^{2}t}{1+k_{2}q_{e}t}$$

(3) $$q_{t}=k_{3}t^{1/2}+B$$

where *k*_1_, *k*_2_, and *k*_3_ represent the rate constants of the kinetic models, *q_t_* is the adsorption capacity of a sample at different time points, and *B* represents the constant of the boundary layer thickness. 

These three adsorption kinetics models were fitted with the experimental data, and the fitting parameters are shown in Table 2. For both the MG and the TH adsorption of BMFH and BMFH/Fe_3_O_4_, the curves of the PSK matched the relationship between the equilibrium adsorption capacities and the equilibrium concentrations better than the curves of the PFK and IPD. The correlation coefficients (*R*^2^) of the PFK were 0.9732–0.9926 and 0.9847–0.9909 for BMFH adsorption of MG and TH, respectively. When the adsorbent was BMFH/Fe_3_O_4_, the correlation coefficients (*R*^2^) of the PFK were 0.9737–0.9862 and 0.9821–0.9912 for MG and TH, respectively. All the *q_e.cat_* values calculated theoretically according to PFK were lower than the experimental *q_e_*, indicating that the adsorption process may not belong to Lagergren’s model. On the contrary, the correlation coefficients (*R*^2^) of the PSK were 0.9949–0.9996 and 0.9966–0.9991 for BMFH and BMFH/Fe_3_O_4_, respectively, when the adsorbate was MG, and 0.9986–0.9998 and 0.9990–0.9999, respectively, when the adsorbate was TH. At the same time, the *q_e.cat_* values calculated theoretically according to PSK were more suitable for the experimental quantification *q_e_*, indicating the applicability and potential advantages of Ho–McKay’s model in the adsorption process; that is, the adsorption rate may be controlled by chemical reactions, and similar reactions that form chemisorption bonds between adsorbent and adsorbent through transfer, exchange, or sharing can promote the adsorption process [23,24,25,26,27]. For the IPD model, the correlation coefficients (*R*^2^) were 0.7582–0.9050 (MG) and 0.7178–0.8271 (TH) for BMFH and 0.7824–0.8741 (MG) and 0.7341–0.8163 (Th) for BMFH/Fe_3_O_4_, demonstrating that the IPD model was not able to explain the whole adsorption process.

#### 2.3.2. Adsorption Isotherm

Adsorption isotherms are often used to study the effect of concentration on adsorption capacity and to help explore how adsorbents interact with adsorbates during the adsorption process [29]. The Langmuir model (Equation (4)) applies to materials with uniform energy adsorption sites and monolayer adsorption layer coverage, and isotherms assume that all adsorption sites are equivalent to uniform surface coverage. In contrast, the Freundlich model (Equation (5)) describes the adsorption process on a non-uniform surface with different energy adsorption sites. They are expressed below:

(4) $$q_{e}=\frac{q_{m}K_{L}C_{e}}{1+K_{L}C_{e}}$$

(5) $$q_{e}=K_{F}C_{e}{}^{1/n_{F}}$$

where *q_m_* (mg/g) represents the maximum adsorption capacity of a sample, *C_e_* (mg/L) is the solution concentration at equilibrium, *K_L_* and *K_F_* represent the constants of the Langmuir and Freundlich models, respectively, and *n_F_* represents the constants of the Freundlich isotherm models.

**Figure 7:**
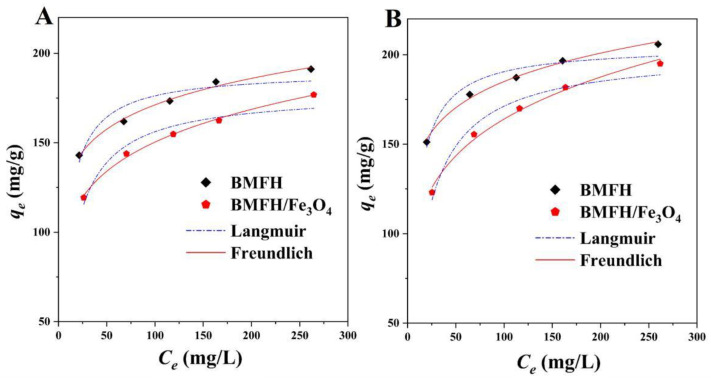
Langmuir and Freundlich isotherm plots of BMFH and BMFH/Fe_3_O_4_ for (**A**) MG and (**B**) TH at 303 K.

In this study, the effect of initial concentrations on the adsorption of MG and TH (50, 100, 150, 200, and 250 mg/L) by BMFH and BMFH/Fe_3_O_4_ are shown in Figure 7, and the data fitted using Langmuir and Freundlich isotherm models are shown in Table 3. With the increase in initial MG and TH concentrations, the adsorption capacities of BMFH and BMFH/Fe_3_O_4_ increased gradually. For BMFH, the correlation coefficients (*R*^2^) of the Langmuir model were 0.8905 for MG and 0.9378 for TH. For BMFH/Fe_3_O_4_, the correlation coefficients (*R*^2^) of the Langmuir model were 0.9380 for MG and 0.9659 for TH. These results indicated that the processes were not homogeneous monolayer adsorption. The correlation coefficients (*R*^2^) of the Freundlich were all higher than 0.99 (0.9928 and 0.9945 of BMFH for MG and TH, 0.9965 and 0.9915 of BMFH/Fe_3_O_4_ for MG and TH, respectively). Furthermore, the intensity factors *n_F_* of BMFH and BMFH/Fe_3_O_4_ related to the adsorption intensity or surface uniformity were 8.3675 and 6.0368 for MG and 8.4253 and 5.1808 for TH, respectively, suggesting that the adsorption process may belong to multilayer adsorption with non-uniform surface [30,31].

#### 2.3.3. Adsorption Thermodynamic Results

Temperature is an important parameter affecting the adsorption process. On the one hand, it can promote the thermal movement of molecules in the reaction system, and on the other hand, it can directly affect the endothermic or exothermic reaction through the thermal energy [27]. The thermodynamic parameters standard entropy (Δ*S*), standard Gibbs free energy (Δ*G*), and standard enthalpy (Δ*H*) were analyzed to study the adsorption process. Δ*H*, Δ*S*, and Δ*G* could be calculated by Equations (6)–(8):

(6) $$\ln\;(K_{T})=\frac{\Delta S}{R}-\frac{\Delta H}{RT}$$

(7) $$K_{T}=\frac{q_{e}}{C_{e}}$$

(8) ΔG=ΔH−TΔS

where *T* is the temperature (K), *K_T_* is the thermodynamic equilibrium constant, and *R* represents the gas constant (8.314 J/K mol).

**Figure 8:**
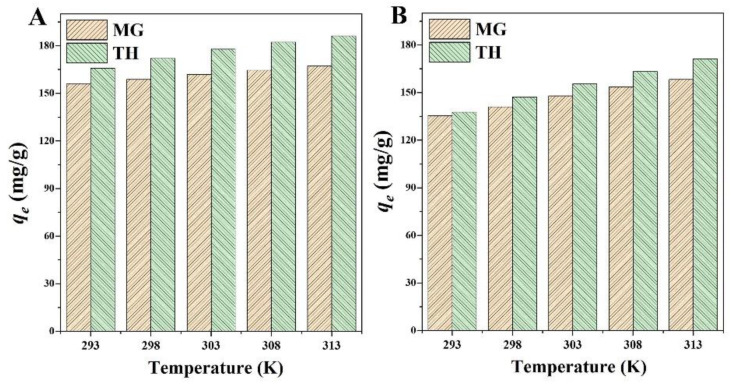
Effect of different temperatures on the adsorption capacities of (**A**) BMFH and (**B**) BMFH/Fe_3_O_4_ for MG and TH.

In this study, the temperature conditions were chosen according to the reported references and our previous work [23,24,25,26,27]. The results of effect of distinct temperatures (293, 298, 303, 308, and 313 K) on the adsorption capacities of the BMFH and BMFH/Fe_3_O_4_ for MG and TH are shown in Figure 8 and the parameters are shown in Table 4. As the temperature increased from 293 to 303 K, the adsorption capacities of BMFH and BMFH/Fe_3_O_4_ for RhB and TH increased. While the temperature further increased to 313 K, the increasing trend of adsorption capacities followed the same pattern. Overtly, the increase in temperature promoted the process of adsorption, and high temperature was conducive to the adsorption of MG and TH by BMFH and BMFH/ Fe_3_O_4_. In addition, it is worth noting that the adsorption capacities of BMFH increased by 11.14 mg/g (156.12 to 167.26 mg/g) for MG and 20.36 mg/g (165.79 to 186.15 mg/g) for TH as the temperature increased from 293 to 313 K; in the meantime, the adsorption capacities of BMFH/Fe_3_O_4_ increased by 22.90 mg/g (135.35 to 158.25 mg/g) for MG and 33.71 mg/g (137.55 to 171.26 mg/g) for TH. These results indicate that the temperature effect was higher on the BMFH/Fe_3_O_4_ than on the BMFH. The ΔG and ΔH were negative and positive, indicating that adsorption is a spontaneous endothermic process [31]. The ΔS (20.06 and 30.80 J mol^−1^ K^−1^ of BMFH for MG and TH, 33.92 and 46.53 J mol^−1^ K^−1^ of BMFH/Fe_3_O_4_ for MG and TH, respectively) were all positive, indicating that the confusion and randomness of the interface between adsorbents and adsorbates increased with the increase in temperature [29,30].

### 2.4. Effect of pH

**Figure 9:**
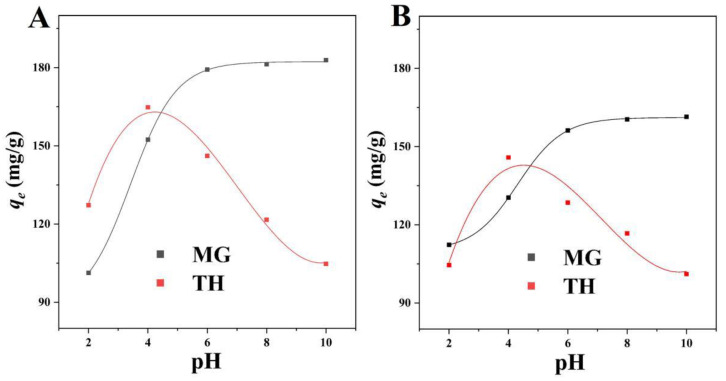
Effect of pH on the adsorption capacities of (**A**) BMFH and (**B**) BMFH/Fe_3_O_4_ for MG and TH.

The pH value is one of the most important factors affecting the adsorption process, mainly by changing the surface properties of adsorbent and adsorbate chemical properties to promote or inhibit the adsorption process [30]. The results of the effect of pH change on the adsorption of MG and TH for BMFH and BMFH/Fe_3_O_4_ in the pH value range from 2 to 10 are shown in Figure 9.

For both BMFH and BMFH/Fe_3_O_4_, the adsorption capacities of MG increased with the increase in pH (6 ≥ pH ≥ 2) and tended to be stable (pH ≥ 6). For TH, the adsorption properties of BMFH and BMFH/Fe_3_O_4_ generally increased first (4 ≥ pH ≥ 2) and then decreased (pH ≥ 4). This can be explained by the existence of TH in different forms at different pH levels (TH^+^ at pH ≤ 3.30, TH^0^ at pH = 3.30–7.68, TH^−^ and TH^2−^ at pH ≥ 7.68) [29]. When the pH was 6, the maximum adsorption capacities of BMFH and BMFH/Fe_3_O_4_ for MG were 179.24 and 156.20 mg/g, respectively; when the pH was 4, the maximum adsorption capacities for TH were 168.79 and 145.81 mg/g, respectively. Electrostatic attraction may play a role in this process; for example, higher pH results in more negative charges on BMFH and BMFH/Fe_3_O_4_ surfaces, which increases the adsorption capacity of positively charged cationic dye MG and decreases the adsorption capacity of the negatively charged form of TH.

### 2.5. Fenton-like Catalysis Performance Results

#### 2.5.1. Effect of BMFH/Fe_3_O_4_ and H_2_O_2_ on Catalytic Performance

**Figure 10:**
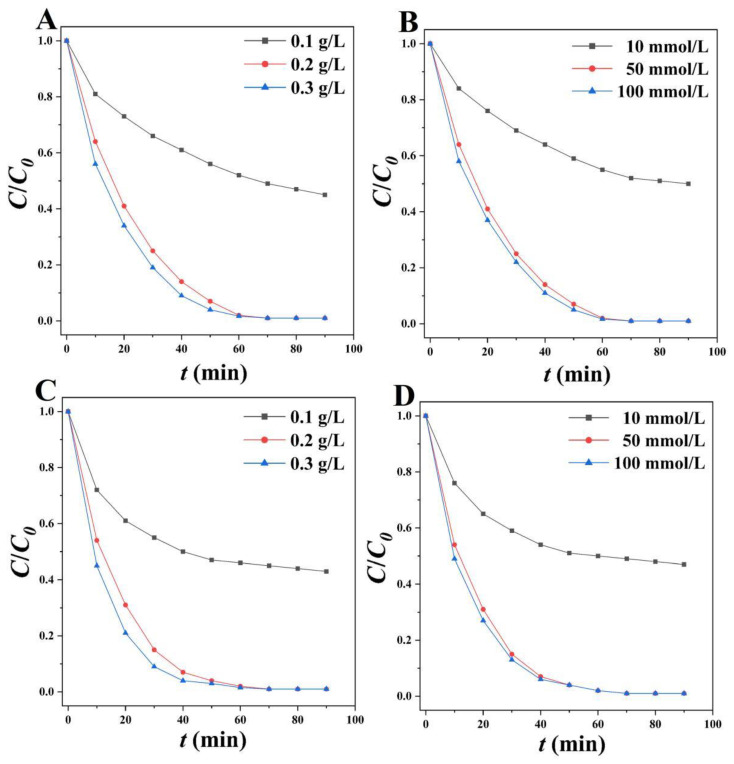
Effect of dosage of BMFH/Fe_3_O_4_ and concentration of H_2_O_2_ on the removal efficiency of (**A**,**B**) MG and (**C**,**D**) TH.

The Fenton-like catalytic performance of BMFH/Fe_3_O_4_ on organic pollutants (MG and TH) was evaluated by control experiments, and the effect of dosage of BMFH/Fe_3_O_4_ and concentration of H_2_O_2_ on the degradation of MG and TH is shown in Figure 10 (A and B for MG, C and D for TH). While the *C*_0_ was 50 mg/L, the concentration of H_2_O_2_ was 50 mmol/L, the pH was 6, and the T was 303 K; the effect of the dosage of BMFH/Fe_3_O_4_ on the removal efficiency of MG was studied. With the increase in BMFH/Fe_3_O_4_ dosage from 0.1 g/L to 0.2 g/L, the removal rate of MG increased from 55% to 99% within 60 min. This is mainly because the higher the amount of BMFH/Fe_3_O_4_ added, the more catalyst active sites provided, and the higher the probability of collision between H_2_O_2_ and the catalyst active sites [19,20]. Therefore, producing more hydroxyl radicals will increase the degradation rate. However, when the amount of BMFH/Fe_3_O_4_ was further increased (0.3 g/L), the removal efficiency of MG was not significantly improved, which was due to the saturation of the active site [19]. Using a dosage of 0.2 g/L of BMFH/Fe_3_O_4_, the effect of H_2_O_2_ concentration on the removal efficiency of MG was studied. When the concentration of H_2_O_2_ increased from 10 mmol/L to 50 mmol/L, the removal efficiency of MG increased from 50% to 99% within 60 min. In a Fenton-like oxidation catalytic reaction system, H_2_O_2_ is the main source of hydroxyl radicals [21]. Increasing the concentration of H_2_O_2_ can enhance the accessibility of H_2_O_2_ to the active site, which is conducive to the generation of active species, which improves the catalytic degradation rate [22]. However, further increase in the concentration of H_2_O_2_ (100 mmol/L) did not provide significant improvement because the BMFH/Fe_3_O_4_ catalytic active site was saturated. Therefore, when the *C*_0_ of MG was 50 mg/L, the optimal ratio of BMFH/Fe_3_O_4_ and H_2_O_2_ was 50 mmol/L and 0.2 g/L, respectively. When the *C*_0_ of TH was 50 mg/L, the pH was 4, and the T was 303 K; when the dosage of BMFH/Fe_3_O_4_ was 0.2 g/L, the concentration of H_2_O_2_ was 50 mmol/L, and the removal efficiency was 99% within 60 min.

#### 2.5.2. Oxidative Radicals Quenching Experiment Results

**Figure 11:**
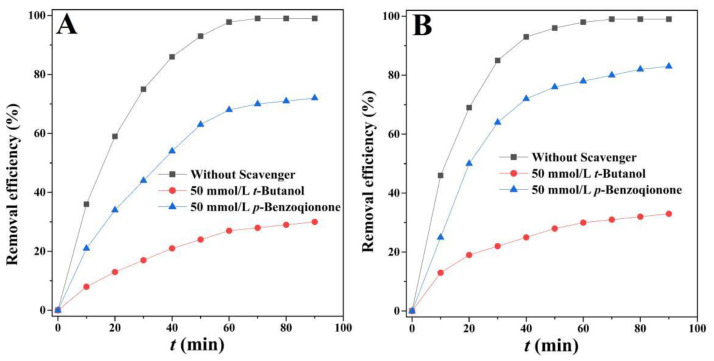
Effect of radical scavenger on the removal efficiency of (**A**) MG and (**B**) TH.

Oxidative radicals produced by H_2_O_2_ play an important role in the catalytic degradation of organic pollutants (MG and TH). The catalyst used to activate H_2_O_2_ has a great influence on the types of radical. *t*-Butanol and *p*-Benzoquinone were used as scavengers of OH∙ and HO_2_∙ to quench the active substances to determine the major oxidative radicals formed in the Fenton-like catalytic reactions [19,20]. The effect of radical scavengers on the removal efficiency of MG and TH are shown in Figure 11. The removal efficiency of both MG and TH was 99% within 60 min without scavenger, while it decreased to only 30% for MG and 33% for TH within 60 min in the presence of 50 mmol/L *t*-Butanol. After adding 50 mmol/L of *p*-Benzoquinone, the removal efficiency of MG and TH was 72% and 83%, respectively. It can be inferred that the ∙OH and HO_2_∙ radicals were generated in the Fenton-like catalytic system of BMFH/Fe_3_O_4_ and H_2_O_2_. In the oxidative catalysis degradation of MG and TH, the ∙OH radical may play a more important role compared with HO_2_∙ [19,20]. 

### 2.6. Cycling Stability and Performance Comparison of BMFH and BMFH/Fe_3_O_4_

**Figure 12:**
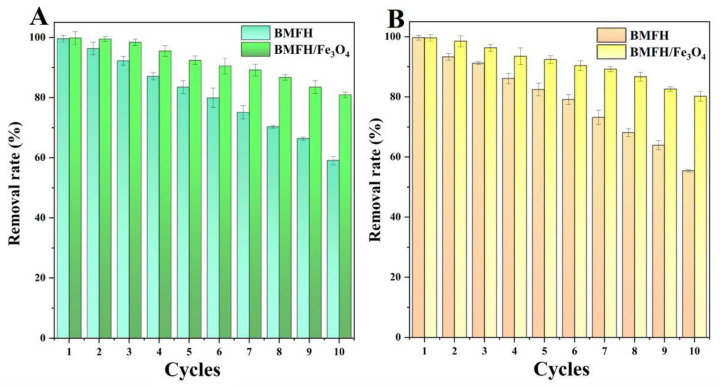
Cycling stability of the BMFH and BMFH/Fe_3_O_4_ to remove (**A**) MG and (**B**) TH.

Cycling stability is very important for evaluating the effectiveness of biochar so that the environmental material can be used repeatedly [29,30]. Compared with BMFH, BMFH/Fe_3_O_4_ could be easily separated from the pollutant water by a magnet due to the magnetic property of the nano-particles of Fe_3_O_4_. In this investigation, 10 cycle experiments were used to assess regenerative capacities of BMFH and BMFH/Fe_3_O_4_ (Figure 12). With the cycles increased the removal rate of BMFH decreased clearly and could only be maintained at 59.1% for MG and 55.4% for TH after 10 cycles. Meanwhile, the removal rate of BMFH/Fe_3_O_4_ could still be maintained above 80% after 10 cycles in the experiment (80.9% for MG and 80.2% for TH). This can be explained by the fact that every time high-temperature carbonization occurs in the regeneration process, it makes the biochar brittle and more fragile, thus affecting its subsequent cycle testing performance. Compared with BMFH, the presence of Fe_3_O_4_ particles may provide a more stable support for BMFH/Fe_3_O_4_ in the process of recycling and regeneration. On the basis of the above data, it can be concluded that BMFH and BMFH/Fe_3_O_4_ both have high stability and great potential for the control of organic pollutants in water environments.

### 2.7. Comparison with Other Biochars

It can be seen from Table S2 (Supplementary Material) that different biochars have different adsorption capacities for organic pollutants (MG and TH). According to the current data, the adsorption capacity of magnetic biochar BMFH/Fe_3_O_4_ (171.26 mg/g) was slightly lower than that of BMFH (186.15 mg/g), but compared with most biochar adsorbents [35,36,37,38,39,40,41,42,43,44,45,46,47,48,49,50,51,52,53,54], BMFH and BMFH/Fe_3_O_4_ still showed good adsorption performance for the removal of MG and TH. We speculate that, despite their low surface area, their unique 3D network structure and rich surface chemistry may play a major role. This result also indicates that not only the specific surface area but also the surface chemistry and spatial structure of biochar may affect the adsorption process. We will explore this further in a follow-up study. Due to the presence of Fe_3_O_4_, BMFH/Fe_3_O_4_ also has the ability of Fenton-like catalytic degradation of organic pollutants (MG and TH). Compared with some catalysts (Table S3) [55,56,57,58,59,60,61,62,63,64], although there is still a certain gap in catalytic performance, it is enough to demonstrate the potential of magnetic biochars prepared by one-step carbonization synthesis in the control of organic pollutants.

### 2.8. Possible Mechanisms

**Figure 13:**
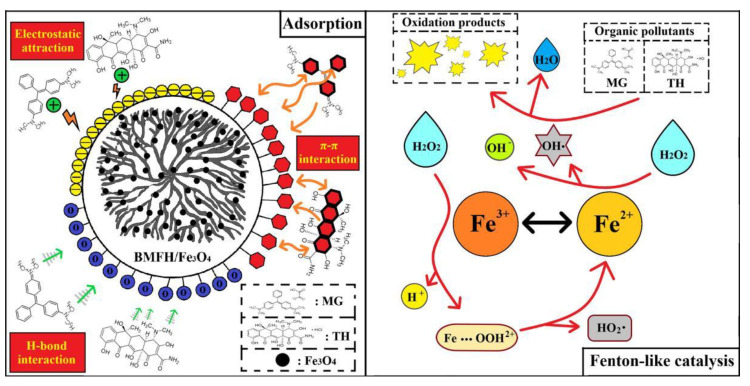
Possible mechanisms of BMFH/Fe_3_O_4_ for removal of MG and TH via adsorption and Fenton-like catalysis.

In this study, BMFH/Fe_3_O_4_ removes organic pollutants (MG and TH) via adsorption and the Fenton-like catalytic method; therefore, the possible mechanisms can be divided into two types, adsorption mechanism and catalytic reaction mechanism (Figure 13).

The possible mechanism in the adsorption process may include electrostatic attraction, H-bond interaction, and π-π interaction. There is a strong electrostatic interaction between organic pollutants and BMFH/Fe_3_O_4_ with negative zeta potential, which may be beneficial to adsorption in a water environment with higher pH. It can be found from FT-IR and XPS spectroscopy test results that there are various functional groups on the surface of BMFH/Fe_3_O_4_ that are conducive to adsorption; for example, the C-O group may form an H-bond with the C-H group in organic pollutants, and the O-H group may form an H-bond with N^+^ or -O- in organic pollutants. FT-IR and Raman spectra indicated that the aromatic rings in organic pollutants may interact π-π with aromatic rings in BMFH/Fe_3_O_4_, thus enhancing the adsorption capacity.

In BMFH/Fe_3_O_4_ synthesized by the one-step method, ferric ions exist in two oxidation states, Fe^3+^ and Fe^2+^, which was also confirmed by XPS results. During the Fenton-like catalytic reaction process, H_2_O_2_ will react with Fe^3+^ to form peroxo-intermediate. Then, peroxo-intermediate will generate HO_2_∙ and Fe^2+^, which will happen again on reacting with the H_2_O_2_ molecule to form OH∙. Finally, OH∙ is involved in catalytic degradation of organic pollutants (MG and TH) to oxidation products. Thus, the best-proposed mechanism is as follows (Equations (9)–(12)):

(9) $${\mathrm{Fe}}^{3+}+H_{2}O_{2}↔Fe\cdots OOH^{2+}+H^{+}$$

(10) $$\mathrm{Fe}\cdots OOH^{2+}\to HO_{2}\cdot +Fe^{2+}$$

(11) $$H_{2}O_{2}+Fe^{2+}\to OH\cdot +OH^{-}+Fe^{3+}$$

(12) $$\mathrm{OH}\cdot +\mathrm{Pollutants}\to \mathrm{Products}+H_{2}O$$

## 3. Materials and Methods

### 3.1. Materials and Reagents

*T. reesei* was extracted and preserved from decayed straw by the Key Laboratory of Straw Comprehensive Utilization and Black Soil Conservation, Ministry of Education, China.

The potato was bought from the local vegetable market. D-glucose monohydrate, FeCl_3_, HCl, H_2_O_2_, and NaOH were supplied by Beijing Chemical Works (Beijing, China). Malachite green (MG), tetracycline hydrochloride (TH), *t*-Butanol, and *p*-Benzoquinone were supplied by Aladdin Chemical (Shanghai) Co., Ltd. (Shanghai, China). All the above reagents were of analytical purity grade and did not require further purification. The water involved in the rinsing and preparation process was deionized water.

### 3.2. Preparation of BMFH/Fe_3_O_4_

*T. reesei* was seeded onto potato-glucose-agar (PDA, 200.0 g/L of potato extract, 20.0 g/L of D-glucose, and 20.0 g/L of agar) solid medium and cultured at 29 °C for 96 h. They were then isolated from PDA plates and inoculated into 200.0 mL potato-glucose-liquid (PDL, PDA medium without agar) medium for growth, resulting in the formation of numerous FH. After incubation for 72 h in a constant temperature incubator at 29 °C, different concentrations of FeCl_3_ (0.001–0.5 mol/L) were added and incubated for another 24 h. After separation from the medium, it was washed with deionized water to remove water-soluble impurities and excess ferric chloride, denoted as FH/Fe^3+^, for subsequent high-temperature experiments. 

FH/Fe^3+^ was calcined under nitrogen atmosphere protection in a horizontal tube furnace. To synthesize magnetic FH biochar materials (BMFH/Fe_3_O_4_) in one step, we divided the calcination process into three temperatures: 100 °C for 30 min, 200 °C for 30 min, and 600 °C for 60 min (Figure S1, Supplementary Materials). The abbreviations BMFH/Fe_3_O_4_-0.001 and BMFH represent the magnetic biochar material from FH/Fe^3+^ containing 0.001 mol/L FeCl_3_ by a one-step calcination method and FH-based biochar, respectively. Biochar yield (BY, %) is a valuable parameter used to evaluate the practicability of production methods, which is calculated by Equation (13):

(13) $$\mathrm{BY}=\frac{M_{biomass}}{M_{biochar}}\times 100\%$$

where *M_biomass_* (g) and *M_biochar_* (g) represent the mass of biomass used for preparation and the mass of obtained biochar, respectively. The results are shown in Table S4 (Supplementary Materials). The BYs of BMFH and BMFH/Fe_3_O_4_-0.001 were 43.85 ± 0.55% and 45.54 ± 1.13%, respectively, indicating the feasibility of this method for biochar production.

### 3.3. Adsorption Performances

In a batch adsorption experiment, 0.20 g/L of BMFH or BMFH/Fe_3_O_4_ was added to a flask containing organic pollutant solutions (MG or TH); the structural formulas of MG and TH are shown in Figure S2 (Supplementary Materials). When the adsorption process reached equilibrium, 1.0 mL of the suspension was taken out, and BMFH or BMFH/Fe_3_O_4_ was filtered through a 0.22 µm filter film. Next, the suspension was centrifuged at 12,000 RPM for 10 min and the supernatant was diluted with deionized water. The concentration of the solution was determined by Agilent Cary-300 UV-vis spectrophotometer. The adsorption capacities of BMFH/Fe_3_O_4_ were calculated by Equation (14):

(14) $$q_{e}=\frac{(C_{0}-C_{e})\times V}{M}$$

where *q_e_* (mg/g) is the adsorption capacity of samples; *C_e_* and *C*_0_ (mg/L) denote the equilibrium and initial concentrations of the dye solution, respectively; and *M* (g) and *V* (L) denote the mass of the samples and the volume of the solutions, respectively.

#### 3.3.1. Adsorption Kinetic Performances

The organic pollutant solutions were prepared at different concentrations (50, 100, and 200 mg/L). Then, 0.2 g/L of BMFH or BMFH/Fe_3_O_4_ was dispersed into flasks containing MG or TH solutions and shaken at 150 RPM in the dark at 303 K. Finally, the concentrations of the solutions were determined at preset time intervals.

#### 3.3.2. Adsorption Isotherm Experiments

The organic pollutant solutions at different initial concentrations (50, 100, 150, 200, and 250 mg/L) were prepared and used to test the adsorption isotherm at 303 K. After adsorption saturation, the absorbance of the solutions was measured by UV-vis spectrophotometer

#### 3.3.3. Adsorption Thermodynamic Experiments

The effect of temperatures (293, 298, 303, 308, and 313 K) on the adsorption capacity of the adsorbates was investigated at an initial concentration of 100 mg/L with 0.2 g/L of BMFH or BMFH/Fe_3_O_4_.

#### 3.3.4. The Effect of pH on Adsorption Capacities

The variation of the adsorption capacity of the samples with pH (2, 4, 6, 8, and 10) was also investigated. The solutions were adjusted to different pH values by HCl and NaOH.

### 3.4. Fenton-like Catalysis Performances

#### 3.4.1. Catalytic Activity Test of BMFH/Fe_3_O_4_

In a batch adsorption experiment, BMFH/Fe_3_O_4_ (0.1, 0.2, and 0.3 g/L) was added to a flask containing organic pollutant solutions (MG or TH). The flask was placed in a constant temperature shaker at 150 RPM. After the adsorption process reached equilibrium, H_2_O_2_ (10, 50, and 100 mmol/L) was added to the flask to initiate the catalysis degradation reaction at 303 K. At regular intervals, 1.0 mL of the suspension was taken out, and BMFH/Fe_3_O_4_ was separated by using a magnet and filtered through a 0.22 µm filter film. After that, the suspension of organic pollutants was centrifuged at 12,000 RPM for 10 min, and the supernatant was diluted with deionized water. Then, the concentration of the solutions was determined by using an Agilent Cary-300 UV-vis spectrophotometer. The removal efficiency (*R*%) of organic pollutants can be determined by using Equation (15):

(15) $$R\%=\frac{C_{0}-C}{C_{0}}\times 100\%$$

where *C*_0_ and *C* are the initial and non-degraded adsorbates concentrations (mg/L), respectively, in the supernatant after centrifuging.

#### 3.4.2. Oxidative Radicals Quenching Experiments

At the beginning of the catalysis experiment, 50 mmol/L of *t*-Butanol or *p*-Benzoquinone was added to study the effect of different oxidation radicals. The concentration of the solutions was determined by using an Agilent Cary-300 UV-vis spectrophotometer.

### 3.5. Cycling Stability Studies

A total of 1.0 g/L of BMFH/Fe_3_O_4_ was added to 100 mL of contaminants in each cycle (100 mg/L). When the experiments finished, the recycled samples were collected by a magnet. After that, the BMFH/Fe_3_O_4_ was washed with water and carbonized for 60 min at 600 °C under nitrogen atmosphere protection. In the next cycle, reused samples were employed as fresh adsorbents. The details of the characterization methods are shown in Supporting Information.

## 4. Conclusions

In this study, magnetic biochar (BMFH/Fe_3_O_4_) with a unique three-dimensional network structure was synthesized by a simple and controllable method, which has both adsorption and Fenton-like catalytic properties. The adsorption capacities of BMFH/Fe_3_O_4_ for organic pollutants (158.2 mg/g for MG and 171.26 mg/g for TH) were higher than those of most biochars. The catalytic degradation of organic pollutants reached 99% in 60 min, which was better than most catalysts. After 10 cycles, the removal ability of BMFH/Fe_3_O_4_ in relation to MG and TH remained above 80%. This work not only prepared magnetic biochar materials with excellent performance, which can better control organic pollutants in water, but more importantly, it also provided a new method for the development of other biomass. In the future, we will continue to explore and optimize this synthesis method and further investigate its role in other biomass (such as agricultural waste).

## Figures and Tables

**Table 1:** The data of N_2_ adsorption–desorption for BMFH/Fe_3_O_4_ prepared under different conditions and BMFH.

Samples	S_BET_ (m^2^/g)	P_m_ (nm)	V_total_ (cm^3^/g)
BMFH	27.91	5.69	0.0369
BMFH/Fe_3_O_4_-0.005	22.77	5.34	0.0236
BMFH/Fe_3_O_4_-0.01	21.59	5.16	0.0214
BMFH/Fe_3_O_4_-0.02	13.40	6.05	0.0202
BMFH/Fe_3_O_4_-0.05	0.89	31.93	0.0071

P_m_ (nm) is the mean pore size and V_total_ (cm^3^/g) is the total pore volume.

**Table 2:** Fitting parameters of adsorption kinetic models for MG and TH at 303 K.

Adsorbates	Adsorbents	Models	Parameters	*C*_0_ (mg L^−1^)		
				50	100	200
MG	BMFH		*q_e_* (mg/g)	142.99	161.89	184.05
		PFK	*k_1_* (min^−1^)	0.2438	0.3179	0.3319
			*q_e.cat_* (mg/g)	134.21	154.69	178.15
			*R* ^2^	0.9732	0.9888	0.9926
		PSK	*k_2_* (g mg^−1^ min^−1^)	0.0031	0.0042	0.0042
			*q_e.cat_* (mg/g)	144.36	162.15	185.38
			*R* ^2^	0.9949	0.9988	0.9996
		IPD	*k_3_* (mg g^−1^ min^−0.5^)	4.4963	3.4926	3.4629
			*B*	98.96	127.57	150.89
			*R* ^2^	0.9050	0.8452	0.7582
	BMFH/Fe_3_O_4_		*q_e_* (mg/g)	119.33	147.90	168.44
		PFK	*k_1_* (min^−1^)	0.2039	0.2208	0.2526
			*q_e.cat_* (mg/g)	112.33	141.29	162.65
			*R* ^2^	0.9737	0.9819	0.9862
		PSK	*k_2_* (g mg^−1^ min^−1^)	0.0029	0.0027	0.0027
			*q_e.cat_* (mg/g)	119.99	150.15	171.13
			*R* ^2^	0.9966	0.9984	0.9991
		IPD	*k_3_* (mg g^−1^ min^−0.5^)	4.2913	4.6966	4.6213
			*B*	77.76	103.57	125.51
			*R* ^2^	0.8741	0.8037	0.7824
TH	BMFH		*q_e_* (mg/g)	151.20	177.81	196.65
		PFK	*k_1_* (min^−1^)	0.2674	0.2241	0.2476
			*q_e.cat_* (mg/g)	144.76	170.19	190.80
			*R* ^2^	0.9847	0.9899	0.9909
		PSK	*k_2_* (g mg^−1^ min^−1^)	0.0034	0.0023	0.0024
			*q_e.cat_* (mg/g)	152.12	179.98	200.38
			*R* ^2^	0.9986	0.9998	0.9996
		IPD	*k_3_* (mg g^−1^ min^−0.5^)	4.0254	5.2583	5.3479
			*B*	112.90	126.09	148.21
			*R* ^2^	0.8271	0.7566	0.7178
	BMFH/Fe_3_O_4_		*q_e_* (mg/g)	123.03	155.47	181.75
		PFK	*k_1_* (min^−1^)	0.1807	0.2642	0.2336
			*q_e.cat_* (mg/g)	116.14	150.24	175.74
			*R* ^2^	0.9821	0.9883	0.9912
		PSK	*k_2_* (g mg^−1^ min^−1^)	0.0024	0.0033	0.0023
			*q_e.cat_* (mg/g)	124.42	157.48	185.32
			*R* ^2^	0.9991	0.9990	0.9999
		IPD	*k_3_* (mg g^−1^ min^−0.5^)	4.6731	3.9191	5.2245
			*B*	77.47	118.65	132.95
			*R* ^2^	0.8163	0.7471	0.7341

**Table 3:** Fitting parameters of adsorption isotherm models for MG and TH at 303 K.

Adsorbates	Adsorbents	Types	Parameters	
MG	BMFH	Langmuir	*q_m_* (mg/g)	190.01
			*K_L_* (L/mg)	0.1279
			*R* ^2^	0.8905
		Freundlich	*K_F_* (mg g^−1^(L mg^−1^)^1/n^)	98.73
			*n_F_*	8.3675
			*R* ^2^	0.9928
	BMFH/Fe_3_O_4_	Langmuir	*q_m_* (mg/g)	178.28
			*K_L_* (L/mg)	0.0707
			*R* ^2^	0.9380
		Freundlich	*K_F_* (mg g^−1^(L mg^-−1^)^1/n^)	70.12
			*n_F_*	6.0368
			*R* ^2^	0.9965
TH	BMFH	Langmuir	*q_m_* (mg/g)	204.94
			*K_L_* (L/mg)	0.1324
			*R* ^2^	0.9378
		Freundlich	*K_F_* (mg g^−1^(L mg^−1^)^1/n^)	107.11
			*n_F_*	8.4253
			*R* ^2^	0.9945
	BMFH/Fe_3_O_4_	Langmuir	*q_m_* (mg/g)	201.47
			*K_L_* (L/mg)	0.0567
			*R* ^2^	0.9659
		Freundlich	*K_F_* (mg g^−1^(L mg^−1^)^1/n^)	67.44
			*n_F_*	5.1808
			*R* ^2^	0.9915

**Table 4:** Fitting adsorption thermodynamic parameters for MG and TH.

Adsorbents	Adsorbates	T (K)	∆*G* (kJ/mol)	∆*H* (kJ/mol)	∆*S* (J mol^−1^ K^−1^)
BMFH	MG	293	−2.00	3.88	20.06
		303	−2.19		
		313	−2.39		
	TH	293	−2.21	6.81	30.80
		303	−2.56		
		313	−2.83		
BMFH/Fe_3_O_4_	MG	293	−1.51	8.43	33.92
		303	−1.87		
		313	−2.18		
	TH	293	−1.56	12.07	46.53
		303	−2.05		
		313	−2.49		

## Data Availability

The study did not report any data.

## Supplementary Materials

The following supporting information can be downloaded at: https://www.mdpi.com/article/10.3390/ijms232012579/s1.
