# Supplementary material for: One-Step Carbonization Synthesis of Magnetic Biochar with 3D Network Structure and Its Application in Organic Pollutant Control
Source: Int J Mol Sci. 2022 Oct 20;23(20):12579. doi: 10.3390/ijms232012579 (PMC9604314; doi:10.3390/ijms232012579)
Supplement: Supplementary file 1 [file ijms-23-12579-s001.zip › ijms-1954306-supplementary.pdf]

One-step carbonization synthesis of magnetic biochar with 3D network structure and its application in organic pollutant control

Xiaoxin Chen[1,2], Jiacheng Lin[1,2], Yingjie Su[1,2,*] and Shanshan Tang[1,2,*]

[1] College of Life Sciences, Jilin Agricultural University, Changchun 130118, China

[2] Key Laboratory of Straw Comprehensive Utilization and Black Soil Conservation, Ministry of Education, Jilin Agricultural University, Changchun 130118, China

[*] Correspondence: yjsu@jlau.edu.cn (Y.S.); tangshanshan81@163.com (S.T.)

S1 Characterization methods

Thermogravimetric analysis of the samples was carried out under the protection of nitrogen flow (TGA, Netzsch STA409PC, Germany). Scanning electron microscopy (SEM, ZEISS SIGMA HD, Germany) and energy dispersive spectrometer (EDS, ZEISS SIGMA HD, Germany) were used to examine the morphology of materials. FT-IR spectrometer was used to characterize the surface functional groups of materials between 400 and 4000 cm^{-1} at a resolution of 1 cm^{-1} (FT-IR, Thermo Fisher Nicolet iS50, USA). X-ray diffraction patterns of the powders were observed by an X-ray diffractometer with a filtered Cu-Ka X-ray source (XRD, Bruker D8 Advance, Germany). Raman spectra of the samples were obtained using a model Renishaw 2000 Raman spectrometer at 514 nm to investigate the presence of defects in the biochar materials. N_2 adsorption-desorption isotherms were used to obtain the porosity of the samples at 77 K (N_2 adsorption-desorption isotherms, Quantachrome Autosorb iQ2, USA). The Brunauer-Emmett-Teller (BET) theory was used to calculate the surface area. The Barrett-Joyner-Halenda (BJH) model was used to analyse the pore size distribution of samples. X-ray photoelectron spectroscopy was used to test the electronic binding energy of the samples (XPS, Thermo Escalab 250Xi+, USA). The zeta potential instrument was used to characterize the surface charge of samples (Zeta potential, Zetasizer Nano ZS90, UK).

Table S1 The element contents of BMFH and BMFH/Fe_3O_4.

Samples	Elements	Mass (%)
BMFH	C	83.16
	O	14.63
	N	2.21
BMFH/Fe_3O_4-0.001	C	81.97
	O	13.67
	N	1.04
	Fe	2.52

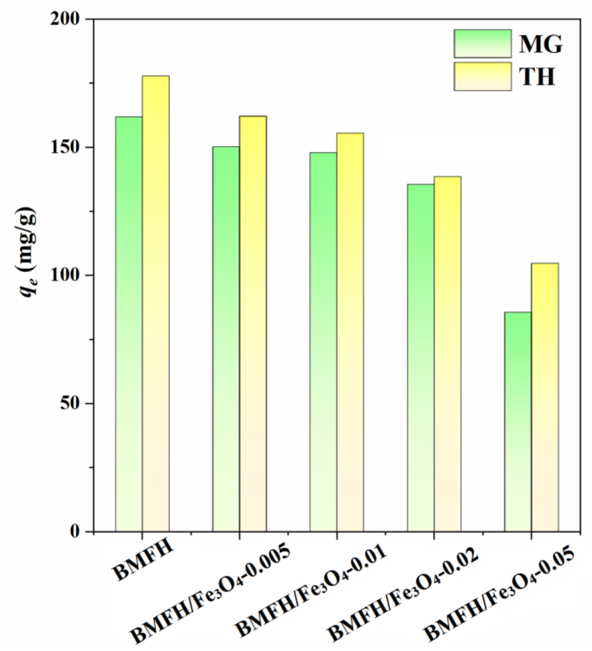

Figure S1. Comparison of the adsorption performance of BMFH/Fe_3O_4 prepared with different Fe^{3+} concentrations and BMFH for MG and TH.

Table S2 Comparison of the adsorption performance of BMFH and BMFH/Fe_3O_4 to MG and TH with other biomass adsorbents.

Adsorbent	S_{BET} (m^2/g)	q_e for MG (mg/g)	q_e for TH (mg/g)	References

BMFH	27.91	167.26	186.15	This work
BMFH/Fe_3O_4	21.59	158.25	171.26	This work
Fan-Palm Biochar (*Livistona chinensis*)	43.25	21.40	-	[35]
Activated charcoa	-	27.00	-	[36]
Sulfur-doped tapioca peel biochar	146.14	30.18	-	[37]
Rice husk biochar	21.70	32.50	-	[38]
Chitosan/activated carbon@UiO-66	108.37	62.10	-	[39]
Rambutan peel-based activated carbon	988.24	87.70	-	[40]
Bio-based activated carbon	173.21	103.64	-	[41]
Pinus roxburghii cone activated carbon	202.00	250.00	-	[42]
Mesoporous rice husk biochar	434.62	373.02	-	[43]
Corn straw-derived biochar	80.10	515.77	-	[44]
Agricultural discarded material biochar	-	-	8.23	[45]
Fe and Mn oxides loaded biochar	2.06	-	14.24	[46]
Bio-char derived from biomass	117.80	-	58.80	[47]
Modified tea-based biochar	409.00	-	84.81	[48]
Pharmaceutical sludge-derived biochar	319.80	-	94.69	[49]
Rice straw derived biochar	115.50	-	98.33	[50]
Fe–N modified rice straw biochar	606.62	-	156.00	[51]
Activated oil palm ash	732.27	-	186.09	[52]
Porous activated carbon from semi-coke	1365.7	-	302.9	[53]

	9		9	
Pharmaceutical sludge porous biochar	675.00	-	379.7<br>8	[54]

Table S3 Comparison of the Fenton-like catalysis performance of BMFH/Fe_3O_4 to MG and TH with other catalysts.

Catalysts	Conditions	Removal efficiency	References
BMFH/Fe_3O_4	Catalysts = 0.2 g/L,<br>C_0 of MG = 50 mg/L,<br>H_2O_2 = 50 mM,<br>pH = 6.0,<br>T = 303 K.	99% in 60 min	This work
BMFH/Fe_3O_4	Catalysts = 0.2 g/L,<br>C_0 of TH = 50 mg/L,<br>H_2O_2 = 50 mM,<br>pH = 4.0,<br>T = 303 K.	99% in 60 min	This work
EDTA-Fe(III)	Catalysts = 500 uM,<br>C_0 of MG = 10 µM,<br>H_2O_2 = 20 mM,<br>pH = 7.0,<br>T = 20 °C.	92.7% in 90 min	[55]
Iron-based nanoparticles from tea extract	Catalysts = 0.74 g/L,<br>C_0 of MG = 50 mg/L,<br>H_2O_2 = 7.4 mM,<br>pH = 4.0,	84.9% in 10 min	[56]

	T = 318 K.		
Ferrous sulfate $FeSO_4$	Catalysts = 10 mM, C_0 of MG = 10 mg/L, H_2O_2 = 25.6 mM, pH = 3.0, T = 40 °C.	93.83% in 60 min	[57]
Iron nanoparticles (Fe NPs)	Catalysts = 11.1 mg/L, C_0 of MG = 50 mg/L, H_2O_2 = 15.6 mM, pH = 6.5, T = 323 K.	98% in 30 min	[58]
Fe_3O_4/graphene aerogels	Catalysts = 0.2 g/L, C_0 of MG = 20 mg/L, Persulfate = 1 mM, pH = 3.0, T = 25 °C.	99% in 20 min	[59]
Core-shell Fe_3O_4@GO-CoPc	Catalysts = 0.4 g/L, C_0 of TH = 40 mg/L, H_2O_2 = 5 mM, pH = 6.0, T = 25 °C.	99% in 180 min	[60]
AC@Fe_3O_4	Catalysts = 0.2 g/L, C_0 of TH = 30 mg/L, Persulfate = 30 mM, pH = 3.0,	99.8% in 180 min	[61]

	T = 25 °C.		
Carbon-bridge-doped g-C_3N_4/Fe_3O_4	Catalysts = 0.5 g/L,<br>*C_0* of TH = 20 mg/L,<br>H_2O_2 = 80 mM,<br>pH = neutral,<br>T = 20 °C.	95.8% in 80 min	[62]
Fe_3O_4 catalyst derived from MIL-88A(Fe)	Catalysts = 0.5 g/L,<br>*C_0* of TH = 10 mg/L,<br>PDS = 1.5 mM,<br>pH = 4.2,<br>T = No mention.	97.5% in 40 min	[63]
FeNi$_3$@SiO$_2$	Catalysts = 0.1 g/L,<br>*C_0* of TH = 10 mg/L,<br>H_2O_2 = 150 mg/L,<br>pH = 7.0,<br>T = 20 °C.	87% in 120 min	[64]

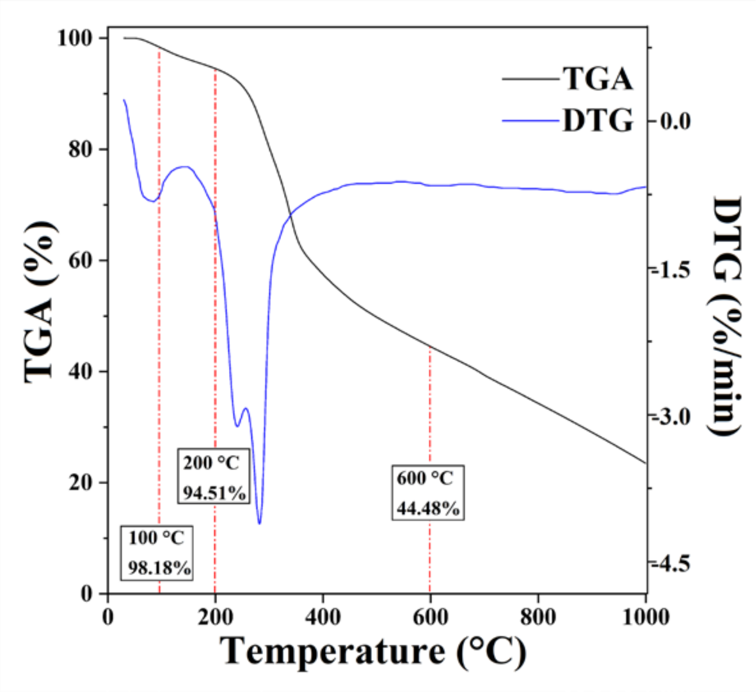

Figure S2. The TGA and DTG curves of FH.

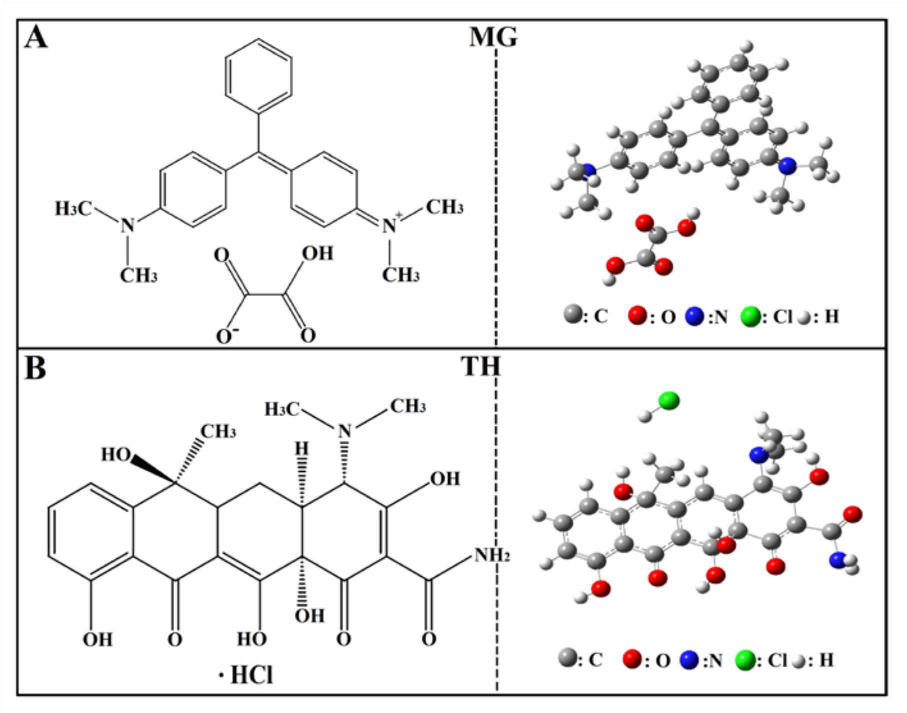

Figure S3. The structural formulas of (A) MG and (B) TH.

Table S4 The *BY%* of BMFH and BMFH/Fe_3O_4.

Samples	*BY* (%)
BMFH	43.85 ± 0.55
BMFH/Fe_3O_4-0.001	45.54 ± 1.13
